# Higher Risk of Anxiety and Depression in Women with Adenomyosis as Compared with Those with Uterine Leiomyoma

**DOI:** 10.3390/jcm11092638

**Published:** 2022-05-07

**Authors:** Ni Li, Ming Yuan, Qiuju Li, Miaomiao Ji, Xue Jiao, Guoyun Wang

**Affiliations:** Department of Obstetrics and Gynecology, Qilu Hospital of Shandong University, Jinan 250012, China; linismile@126.com (N.L.); sddxqlyyym@163.com (M.Y.); qiuju901220@163.com (Q.L.); jmmdoc0506@163.com (M.J.); jiaoxue987@163.com (X.J.)

**Keywords:** adenomyosis, anxiety, depression, quality of life

## Abstract

The main symptoms of adenomyosis may adversely affect physical and mental health and quality of life (QOL). However, studies are few on this topic. This study evaluated the effect of adenomyosis on anxiety, depression, and QOL. Participants with adenomyosis (*n* = 90) or leiomyoma (*n* = 59) completed questionnaires, including the visual analog scale (VAS) for pain, Hospital Anxiety and Depression Scale (HADS), and Short Form (SF)-36. HADS anxiety and depression positive rates, physical (PCS) and mental (MCS) component summary scores, and eight subscale scores of SF-36 were compared between the two groups. Among patients with adenomyosis, the incidence of anxiety symptoms was 28.9% (control group, 10.2%; OR = 3.589, 95% CI: 1.375–9.367), with 10% of patients showing moderate-to-severe symptoms. The incidence of depressive symptoms was 14.4% (control group, 3.4%; OR = 4.812, 95% CI: 1.044–22.168). The case group had significantly lower PCS and MCS scores than the control group. In patients with adenomyosis, being employed (OR = 6.393, 95% CI: 1.153–35.440) and perianal pain (OR = 25.419, 95% CI: 2.504–258.024) were risk factors for anxiety, and perianal pain (OR = 15.208, 95% CI: 3.050–75.836) was a risk factor for depression. Compared with leiomyoma, adenomyosis is associated with a higher risk of anxiety and depression, with a poorer QOL.

## 1. Introduction

Adenomyosis is characterized by uterine enlargement, caused by ectopic endometrial tissue, comprising both glands and stroma, growing deep within the myometrium [1]. It is a common gynecological disease, but its mechanism of action is unclear. Adenomyosis mainly occurs in premenopausal women aged 40–50 years, with a prevalence of 5–70% [2]. In the past, epidemiological data were mainly from patients with hysterectomy. The lack of population-based epidemiological data and inconsistent diagnostic criteria have led to marked changes in disease prevalence. In recent years, with the improvement in magnetic resonance imaging (MRI), two- and three-dimensional transvaginal sonography, and other technologies, the detection of adenomyosis has gradually increased, and it may now be detected at a younger age [3,4,5]. In a United States population-based study (2006–2015), the overall incidence of adenomyosis was 1.03% [6]. Several ultrasonographic criteria have been utilized for the diagnosis of adenomyosis, including uterine enlargement, asymmetry of the anterior and posterior uterine wall thickness, presence of heterogeneous myometrial areas, and poor definition and thickening of the junctional zone (JZ). In an MRI, typical adenomyosis appears as an ill-demarcated low-signal intensity area on T2-weighted images, and thickening of the JZ of the uterus (≥12 mm) [7].

Gureje et al., found a strong and symmetrical relationship between persistent pain and psychological disorders [8]. Iron-deficiency anemia is associated with higher levels of psychological distress [9,10]. An infertility diagnosis is a risk factor for future depression in those undergoing fertility treatments [11,12]. The main symptoms of adenomyosis are progressive dysmenorrhea, chronic pelvic pain, menorrhagia, and infertility, which affect a majority of women of childbearing age [7,13]. Hysterectomy, which is currently the only radical curative method for adenomyosis, is unsuitable for patients who have fertility requirements or wish to preserve their uterus. However, other treatments have shortcomings, such as recurrence and adverse effects. For patients with adenomyosis who undergo conservative treatment, a series of persistent or recurring symptoms, such as dysmenorrhea and menorrhagia, usually have continuous adverse effects on their physical and mental health and quality of life. Compared to patients with uterine myoma, patients with adenomyosis more frequently have a history of depression (up to 57.1%), and their use of antidepressants is also higher [14].

Compared with pain patients without depression or anxiety, pain patients with anxiety or depression, such as those with primary pain, cancer pain, neuropathic pain, and visceral pain, have a worse response to pain treatment, lower satisfaction, more severe and longer-lasting pain symptoms, and reduced pain tolerance [15,16,17]. Drugs and surgery are currently inevitable treatment modalities, but the pain’s etiology cannot be substantially improved in the short term. Psychological evaluation needs to be expanded to improve treatment effects. Investigators have begun to study the impact of adenomyosis on the quality of life of patients and have used indexes to evaluate the effect of treatment [18,19,20]. However, only a few related studies have been conducted in this field. Therefore, this study aimed to evaluate the impact of adenomyosis on anxiety, depression, and quality of life through investigation, to remind gynecologists to pay attention to the psychological state of patients with adenomyosis, provide appropriate treatment, and help in the long-term management of adenomyosis.

## 2. Materials and Methods

**Figure 1:**
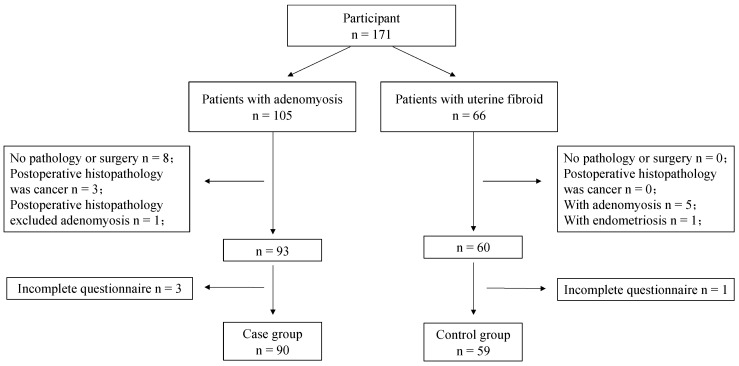
Data collection flowchart. An incomplete questionnaire indicates a patient did not complete the required questionnaire, such as HADS or SF-36.

The study included patients who were hospitalized at the Department of Gynecology, Qilu Hospital of Shandong University (Jinan, China), from 8 June 2019 to 7 January 2020. The case group included 90 patients with adenomyosis diagnosed by histopathology. The inclusion criteria for the case group were as follows: (1) admission to the gynecological ward; (2) main diagnosis of adenomyosis, and (3) provision of informed consent to participate in the study. The exclusion criteria were as follows: (1) a postoperative histopathological diagnosis excluding adenomyosis or a main diagnosis of non-adenomyosis; (2) co-occurrence of malignant tumor; (3) other serious internal and external diseases and serious cognitive problems that could prevent cooperation with the investigation, and (4) poor compliance and refusal to cooperate with investigators. The control group included 59 patients with leiomyoma. The inclusion criteria for the control group were as follows: (1) main diagnosis of leiomyoma; (2) preparation for surgical treatment in the gynecological ward at the same time as the case group, and (3) provision of informed consent to participate in this study. The exclusion criteria were: (1) postoperative histopathological findings complicated with adenomyosis or endometriosis; (2) co-occurrence of malignant tumor; (3) presence of other serious internal and external diseases and serious cognitive problems that could prevent cooperation with the investigation, and (4) poor compliance and refusal to cooperate with investigators. A total of 149 participants were included in the analysis (Figure 1).

### 2.1. Assessments and Questionnaires

All patients completed questionnaires and underwent assessments after admission and before surgical treatment.

#### 2.1.1. Assessment of Anxiety and Depression

Anxiety and depression levels were evaluated using the Hospital Anxiety and Depression Scale (HADS) [21]. This scale is commonly used to screen for anxiety and depression symptoms. This scale includes two subscales, Anxiety (HADS-A) and Depression (HADS-D). The higher the subscale score, the higher the degree of anxiety or depression. The highest possible total HADS-A score is 21. If the score is ≥8, the patient is considered to have anxiety; 0–7 points indicate a normal state, 8–10 points indicate mild anxiety, 11–15 points indicate moderate anxiety, and ≥16 points indicate severe anxiety. The HADS-D scale follows a similar pattern with regard to depression [22]. The Cronbach’s alpha coefficient of the scale in this study was 0.886.

#### 2.1.2. Assessment of Quality of Life

Quality of life was measured using the 36-item Short-Form Health Survey (SF-36) [23]. The SF-36 measures eight health concepts: (1) physiological function (PF); (2) role-physical (RP); (3) bodily pain (BP); (4) general health (GH); (5) vitality (VT); (6) social functioning (SF); (7) role-emotional (RE), and (8) mental health (MH). Scores also contribute to two composite scores: physical component summary (PCS) and mental component summary (MCS). The Cronbach’s alpha coefficient of the scale in this study was 0.916.

#### 2.1.3. Pain Assessment

The visual analog scale (VAS) was used to evaluate the degree of pain during the patient’s last menstruation. We divided VAS into three grades: 0 to 3, no or mild pain; 4 to 6, moderate pain, and 7 to 10, severe pain.

#### 2.1.4. Demographic and Clinical Variables

We used a self-designed questionnaire to obtain information regarding demographic and clinical characteristics, such as age, height, weight, educational level, occupation, smoking history, menstruation, marriage, and childbearing history, past history, operation history, and relevant information on adenomyosis, including clinical symptoms, course of disease, and treatment.

### 2.2. Outcome Measures

The main outcome measures in this study were the comparison of the HADS-A and HADS-D positive rates of the two groups and the PCS and MCS scores in the SF-36 questionnaire. Secondary indicators included the relationship and factors influencing anxiety, depression, and quality of life.

### 2.3. Statistical Analysis

Statistical analysis of the collected data was performed using SPSS version 25 for Mac OS (IBM, Armonk, NY, USA). None of the continuous variables were normally distributed; therefore, they are presented as median (interquartile range). Data analysis of continuous variables and ordinal categorical variables was performed using the Wilcoxon rank-sum test. Data analysis of non-ordinal categorical variables was performed using the Chi-square test. Logistic regression analysis was used to identify the risk factors. Spearman’s correlation test was used for the correlation analysis. Statistical significance was set at *p* < 0.05.

## 3. Results

### 3.1. Demographic and Clinical Characteristics

In the case group, the median age was 44 years, and 57.8% of the patients were aged 40–49 years. The body mass index (BMI) was 24.4 (22.2–27.3) kg/m^2^, and 56.2% of patients were overweight (36%) or obese (20.2%). In the case group, 84.4% had a history of uterine surgery, 43.3% had a history of uterine cavity surgery, and 22.2% had a history of ovarian and fallopian tube surgery. Apart from a history of surgery, there were no significant differences between the two groups in terms of age, BMI, educational background, employment, smoking history, age at menarche, or parity (Table 1).

The VAS score of patients with adenomyosis was 9 (6–10) (controls: 0 (0–5.2), *p* < 0.001)), and 81.1% of the case group had moderate-to-severe pain. When they experienced pain, 65.6% of patients required analgesics to relieve their symptoms. Among the case group, 32.8% had hypermenorrhea. The hemoglobin level in the case group was 110 (92–123) g/L, and 21.8% had moderate-to-severe anemia. There were no significant differences in infertility, hypermenorrhea, or anemia between the two groups (Table 2).

### 3.2. Anxiety and Depression

Nine of the patients with adenomyosis were diagnosed with anxiety or depression in the past, and four of these patients received psychotherapy. No significant differences were found between the two groups regarding the history of anxiety, depression, or treatment (Table 3).

**Figure 2:**
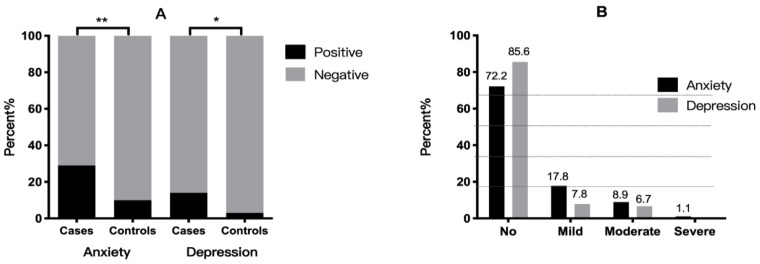
(**A**): Distribution of anxiety and depression. * *p* < 0.05, ** *p* < 0.01. (**B**): Severity of anxiety and depression in patients with adenomyosis.

The HADS-A scores revealed that 28.9% (*n* = 26) of the case group presented with anxiety, which was moderate to severe in 10% (*n* = 9). In the control group, the HADS-A scores revealed that 10.2% (*n* = 6) of women presented with anxiety, which was moderate to severe in 5.1% (*n* = 3). There was a significant difference in the rate of anxiety symptoms between the two groups (X^2^ = 7.405, *p* = 0.007; OR = 3.589, 95% CI: 1.375–9.367), and there were more people with moderate-to-severe anxiety in the case group (Figure 2).

In the case group, the HADS-D scores revealed that 14.4% (*n* = 13) had depressive symptoms, which were moderate to severe in 6.7% (*n* = 6). The HADS-D scores revealed that 3.4% (*n* = 2) of the control group presented with depression, which was moderate to severe in 1.7% (*n* = 1). There was a significant difference in the rate of depression symptoms between the two groups (X^2^ = 4.810, *p* = 0.028; OR = 4.812, 95% CI: 1.044–22.168), and there were more people with moderate-to-severe anxiety in the case group (Figure 2).

Univariate logistic regression analysis was used to identify risk factors for anxiety in patients with adenomyosis. Variables with *p* < 0.1 (employment, dyspareunia, and perianal pain) were introduced into the multivariate logistic regression analysis to determine independent risk factors for anxiety in patients with adenomyosis. Being employed (OR = 6.393, 95% CI: 1.153–35.440) and perianal pain (OR = 25.419, 95% CI: 2.504–258.024) were identified as risk factors for anxiety in patients with adenomyosis.

Factors identified as significantly different between the case and control groups underwent univariate binary logistic regression to analyze the risk factors for depression in patients with adenomyosis. Only the *p*-value for perianal pain was less than 0.1; therefore, multivariate logistic regression analysis was not performed. Perianal pain (OR = 15.208, 95% CI: 3.050–75.836) was identified as a risk factor for depression in patients with adenomyosis.

### 3.3. Quality of Life

**Figure 3:**
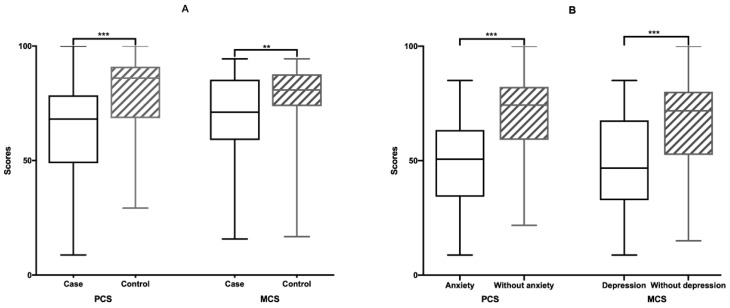
SF-36 ** *p* < 0.01, *** *p* < 0.001. (**A**) SF-36 score of patients with adenomyosis and leiomyoma. (**B**) SF-36 score of anxiety and depression in patients with adenomyosis.

Women with adenomyosis had lower scores than controls for both PCS (case: 68.125 (49–78.5) vs. controls: 86 (68.5–91), *p* < 0.001) and MCS (case: 71.1 (58.95–84.5) vs. control: 80.8 (73.8–87.7), *p* = 0.002) scores. Except for PF and MH, participants with adenomyosis had lower scores in the other SF-36 health subscales (Table 4 and Figure 3A).

### 3.4. Relationship between Anxiety, Depression, and Quality of Life in Patients with Adenomyosis

There was a significant correlation between anxiety and depression (r = 0.568, *p* < 0.001) in the case group. In this study, 13.3% (*n* = 12) of patients with adenomyosis had both anxiety and depression symptoms. Spearman’s correlation revealed that PCS was negatively correlated with HADS-A scores (r_s_ = −0.454, *p* < 0.001) and HADS-D scores (r_s_ = −0.439, *p* < 0.001), and MCS was also negatively correlated with HADS-A scores (r_s_ = −0.653, *p* < 0.001) and HADS-D scores (r_s_ = −0.676, *p* < 0.001) (Table 5). There was a significant correlation between quality of life, anxiety, and depression. Patients with symptoms of anxiety and depression tended to have a lower quality of life (Figure 3B). Similarly, patients with a low quality of life were more likely to suffer from anxiety and depression.

## 4. Discussion

In this study, women with adenomyosis had higher scores in the anxiety and depression subscales of the HADS, as well as lower scores in all domains and in the PCS and MCS scores of the SF-36 questionnaire, compared to women with leiomyoma. This result is consistent with the study of Alcalde et al., on outpatients with adenomyosis [18]. This phenomenon also exists in patients with other chronic diseases, such as diabetes, cardiovascular disease, and rheumatoid arthritis [24]. However, a previous study in the United States showed that 57.1% of patients with adenomyosis had a history of depression [14], which is notably higher than in this study. This may be because the prevalence of depression in the US population is higher than that in the Chinese population (19.2% vs. 6.5%) [25], and the Chinese have insufficient awareness of mental illness and are unwilling to admit to having mental problems [26].

The endocannabinoid system (ECS) is a neuromodulatory system that can coordinate appropriate behavioral responses, which are essential for long-term survival and the health of the body. Disorders in ECS signal transduction can lead to negative emotional states, such as anxiety and depression [27,28,29]. In women with adenomyosis, cannabinoid receptor 1 expression is downregulated [30]. Therefore, it is speculated that a disorder of the ECS may be the main reason for the increased prevalence of anxiety in patients with adenomyosis. Xu et al., suggested that sympathetic-nerve-derived neurotransmitters, such as noradrenaline, may promote the development of adenomyosis through activation of their respective receptors on adenomyotic lesions [31]. Activation of peripheral presynaptic CB(1) receptors inhibits noradrenaline release from sympathetic nerve terminals [32]. The activation of cannabinoid receptors can reduce anxiety, regulate mood, inhibit the development of adenomyosis, and may be a potential target for the treatment of adenomyosis.

Current evidence indicates that women with endometriosis have an increased prevalence of psychological disorders that correlate more with pain itself than with endometriosis per se [33,34,35]. In the present study, quality of life and psychological well-being were not found to be related to the severity of pain. However, Alcalde et al., found that when it is associated with symptoms, the quality of life is further diminished [18]. It has not yet been elucidated whether depression and anxiety determine an increased perception of pain or whether pain causes psychopathological symptoms. However, anxiety and depression could increase pain perception, both emotionally and cognitively, leading to less tolerance to pain and greater sensitivity to physical sensations in general [36]. In our research, it was found that perianal pain is a risk factor for anxiety and depression in patients with adenomyosis. Patients with adenomyosis with perianal pain were more likely to have symptoms of anxiety and depression. Compared with unemployed people, employed patients with adenomyosis were more likely to have symptoms of anxiety. This is because adenomyosis has a clinically relevant impact on work productivity, with higher rates of absenteeism, overall loss of work productivity, and impairment of daily activities [18]. In this study, most patients with anxiety and depression that were screened with the questionnaire did not realize they had anxiety and depression, and most Chinese people are unwilling to see a psychologist [26], which explains why the number of patients with adenomyosis who were previously diagnosed with anxiety or depression was low in the study. Studies have shown that the physical symptoms of chronic disease are significantly higher in patients with anxiety or depression than in those without anxiety and depression and that physical symptoms can be significantly improved after treatment for anxiety and depression [24,37,38]. Anxiety symptoms affect the outcome of pain treatment. Even if analgesics are used, patients with anxiety symptoms are still less satisfied with pain treatments [17]. Research shows that improving the mental health status of patients with chronic diseases can improve their quality of life and improve the treatment effect of diseases [37,38]. Zhao et al., suggests that progressive muscle relaxation training is effective in improving anxiety, depression and QOL in endometriosis patients under GnRH agonist therapy [39]. Considering the high incidence of anxiety and depression in patients with adenomyosis and that both conditions reduce the quality of life of patients, thus, influencing the effect of treatments, attention should be paid to the mental problems of patients, especially those with perianal pain and employed during clinical diagnosis and treatment. Patients with adenomyosis can be screened through a simple scale to identify the symptoms of anxiety and depression early and provide patients with personalized treatment and necessary psychotherapy to improve the treatment and long-term management of adenomyosis. This study found that the quality of life of patients with adenomyosis was poor, both in PCS and MCS, which was similar to previous studies [18,40]. Alcalde et al., compared 89 patients with adenomyosis with 203 normal women and found that the eight dimensions of the SF-36 scale in the observation group were significantly lower than those in the control group. However, in this study, there was no significant difference in PF or MH compared with the control group, which may be related to the different criteria for selecting patients. In this study, the control group comprised patients with leiomyoma, which will also affect the quality of life of the patients [41]. In this study, SF-36 was used to measure the quality of life of the patients because it has universal applicability and met the requirements of this study for comparison with patients with other diseases. However, this scale cannot assess the impact of adenomyosis on the quality of life of patients in terms of sexual life, pregnancy, and response to treatment, which are commonly assessed in other universal scales. The Endometriosis Health Profile (EHP-30), a proprietary scale for endometriosis, provides a comprehensive assessment on the quality of life of patients in daily life, work, sexual relationships, education, social interaction, and psychology [42]. This scale has been used to assess the therapeutic effects of adenomyosis [43]. However, the validity and reliability of this scale in patients with adenomyosis require further research.

This was a cross-sectional study, without longitudinal, dynamic observation, and it could only show the patient’s anxiety, depression, and quality of life at the time of evaluation. To obtain a histopathological diagnosis, the patients selected in this study were only hospitalized patients; however, compared with outpatients, the anxiety, depression, and quality of life of inpatients may have been more severe. Moreover, the sample size of this study was small. To determine the prevalence of anxiety and depression in patients with adenomyosis and the influencing factors, large-sample, multicenter cohort studies are warranted. Further research is needed to improve the mental health of these patients.

## 5. Conclusions

In our study, compared to those with leiomyoma, patients with adenomyosis had a higher risk of anxiety and depression, and the quality of life of these patients was poor. Being employed and perianal pain were risk factors for anxiety in patients with adenomyosis, while perianal pain was the risk factor for depression in patients with adenomyosis. Based on these results, patients with adenomyosis can be screened through a simple scale to identify the symptoms of anxiety and depression early and be provided with individualized treatment and psychotherapy to improve treatment outcomes and the long-term management of adenomyosis.

## Figures and Tables

**Table 1:** Demographic characteristics.

M(P25–75) ^a^/N(%)	Cases (*n* = 90)	Controls (*n* = 103)	X^2^/Z ^b^	*p*
Age (years)	44 (40–48)	44 (36–47)	−1.021	0.307
BMI (kg/m^2^)	24.4 (22.2–27.3)	23.3 (21.4–26.5)	−1.547	0.122
Educational background			1.857	0.395
Primary Education	13 (14.4%)	6 (10.2%)		
Secondary Education	63 (70%)	39 (66.1%)		
Higher Education	14 (15.6%)	14 (23.7%)		
Being employed			0.241	0.623
No	23 (25.6%)	13 (22%)		
Yes	67 (74.4%)	46 (78%)		
Smoking			1.382	0.240
No	86 (97.7%)	60 (100%)		
Yes	2 (2.3%)	0 (0%)		
Age at menarche (Years)	14 (13–15)	14 (12–15)	−0.967	0.334
Prior surgery				
History of uterine cavity operation	76 (84.4%)	42 (71.2%)	3.802	0.051
Prior uterine surgery	39 (43.3%)	15 (25.4%)	4.947	**0.026 ^c^**
Adnex surgery history	20 (22.2%)	4 (6.8%)	6.289	**0.012**
Pelvic and Abdominal Surgery	6 (6.7%)	7 (11.9%)	1.209	0.272
Tubal sterilization	11 (12.2%)	5 (8.5%)	0.522	0.470
Parity	1 (1~2)	1 (1~2)	−0.662	0.508
With endometriosis	15 (16.7%)	-	-	-

^a^: M(P25–75) means median with interquartile range. ^b^: It means X^2^ or Z value. ^c^: Bold values indicate *p* < 0.05.

**Table 2:** Clinical symptoms.

M(P25–75)/N (%)		Cases	Controls	X^2^/Z	*p*
Pain	VAS	9 (6–10)	0 (0–5.2)	−7.666	**<0.001**
Severity				46.026	**<0.001**
	No or mild pain	17 (18.9%)	41 (69.5%)		
	Moderate pain	10 (11.1%)	9 (47.4%)		
	Severe pain	63 (70%)	9 (15.3%)		
Types of Pain	Dysmenorrhea	78 (86.7%)	19 (32.8%)	45.383	**<0.001**
	Chronic pelvic pain	3 (3.3%)	1 (1.7%)	0.347	0.556
	Lumbago	34 (38.2%)	6 (10.2%)	14.137	**<0.001**
	Dyspareunia	6 (6.7%)	0 (0%)	4.146	**0.042**
	Perianal pain	8 (9%)	0 (0%)	5.606	**0.018**
Analgesics		59 (65.6%)	4 (7.0%)	48.830	**<0.001**
Hypermenorrhea		51 (57.3%)	24 (41.4%)	3.563	0.059
Hemoglobin (g/L)		110 (92–123)	116 (101–128)	−1.719	0.086
	Mild anemia	24 (27.6%)	12 (20.3%)		
	Moderate anemia	18 (20.7%)	10 (16.9%)		
	Severe anemia	1 (1.1%)	1 (1.7%)		
Infertility		15 (18.3%)	7 (13.2%)	0.610	0.435

Bold values indicate *p* < 0.05.

**Table 3:** Anxiety and depression in patients with and without adenomyosis.

M(P25–75)/N (%)	Cases	Controls	X^2^/Z	*p*
HADS-A ^a^	5 (2–8)	3 (1–6)	−2.731	**0.006 ^b^**
HADS-D ^c^	3 (1–6)	1 (0–3)	−2.897	**0.004**
History of anxiety and depression	9 (10%)	5 (8.5%)	0.097	0.755
Anxiety and depression treatment history	4 (4.4%)	1 (1.7%)	0.831	0.362

^a^: the score of HADS-anxiety. ^b^: Bold values indicate *p* < 0.05. ^c^: the score of HADS-depression.

**Table 4:** SF-36 in patients with and without adenomyosis.

		Cases		Controls			
		Mean	M(P25–75)	Mean	M(P25–75)	Z	*p*
PCS		64.0	68.125 (49~78.5)	80.2	86 (68.5–91)	−4.980	**<0.001**
	PF	89.2	95 (85–100)	93.7	95 (90–100)	−1.710	0.087
	RP	61.4	75 (0–100)	81.1	100 (75~100)	−3.058	**0.002**
	BP	50.2	51 (22–74)	76.9	80 (62~100)	−4.960	**<0.001**
	GH	55.1	56 (40–72)	68.9	72 (50–87)	−3.469	**0.001**
MCS		68.1	71.1 (58.9–84.5)	78.0	80.8 (73.8–87.7)	−3.068	**0.002**
	VT	67.3	67.5 (55–85)	76.3	80 (65–90)	−2.407	**0.016**
	SF	65.3	77.8 (55.6–77.8)	71.8	77.8 (66.7–77.8)	−2.290	**0.022**
	RE	64.8	83.3 (33.3–100)	84.7	100 (66.7–100)	−3.086	**0.002**
	MH	75.0	80 (60–92)	79.3	84 (72–88)	−1.048	0.295

Bold values indicate *p* < 0.05. PCS: physical component summary. MCS: mental component summary. PF: physiological function. RP: role-physical. BP: bodily pain. GH: general health. VT: vitality. SF: social functioning. RE: role-emotional. MH: mental health.

**Table 5:** Relationship between anxiety, depression, and quality of life in patients with adenomyosis.

	PCS		MCS	
	r_s_	*p*	r_s_	*p*
HADS-A	−0.455	<0.001	−0.654	<0.001
HADS-D	−0.439	<0.001	−0.676	<0.001

PCS: physical component summary. MCS: mental component summary. HADS-A: the Hospital Anxiety and Depression Scale-anxiety scores. HADS-D: the Hospital Anxiety and Depression Scale-depression scores.

## Data Availability

The data presented in this study are available upon reasonable request from the corresponding author.
